# Implementation Outcomes and Lessons From a Health‐Department‐Driven Quality‐Based Financing Approach to HIV Prevention Programmes in New York City, United States: A Mixed Methods Analysis

**DOI:** 10.1002/jia2.70194

**Published:** 2026-10-08

**Authors:** Sarit A. Golub, Ariel M. de Roche, Lila Starbuck, Imani K. Hall, Brittany C. Wilbourn

**Affiliations:** ^1^ Hunter Alliance for Research and Translation Department of Psychology Hunter College of the City University of New York (CUNY) New York City New York USA; ^2^ Einstein‐Rockefeller‐CUNY Center for AIDS Research Albert Einstein College of Medicine Bronx New York USA; ^3^ Department of Psychology Basic and Applied Social Psychology PhD Program Graduate Center of the City University of New York New York City New York USA; ^4^ HIV Research Branch Division of HIV Prevention Centers for Disease Control and Prevention Atlanta Georgia USA

**Keywords:** CFIR (Consolidated Framework for Implementation Research), diffusion of innovations, HIV prevention, implementation outcomes, implementation strategies, mixed methods research

## Abstract

**Introduction:**

In 2021, the New York City Health Department (NYCHD) became one of the first jurisdictions in the United States to adopt a quality‐based financing (QBF) approach to HIV prevention programming, shifting away from the traditional fee‐for‐service model. NYCHD used a multi‐level approach that defined quality through three programme‐level implementation strategies (PLS): universal screening; holistic care provision; and sex‐positive, “risk‐free” HIV prevention conversations. By focusing their QBF programme on implementation strategies in addition to service delivery outcomes, NYCHD held agencies accountable for both *what* was provided and *how* services were delivered.

**Methods:**

Project PACE is a CDC‐funded implementation science (IS) partnership between a public university and the NYCHD designed to document and evaluate their QBF approach. Data collected May 2023–September 2024 include: (1) thematic coding of qualitative interviews with agency staff (*n* = 29); (2) quantitative analysis of staff survey data (*n* = 69); and (3) thematic coding of technical assistance meeting minutes/programme monitoring data (document *n* = 771). This paper presents an analysis of the impact of the strategy on 19 funded agencies, focusing on implementation outcomes and applying the Consolidated Framework for Implementation Research (CFIR) to examine barriers and facilitators to the implementation process.

**Results:**

The quality‐focused implementation strategies were rated as highly acceptable, feasible and appropriate. Agencies reported multiple relative advantages of quality‐focused implementation: more holistic care, increased access to services, integration of mental health and sexually transmitted infection testing into HIV prevention, reduced stigma and improved client rapport. Acceptability and feasibility ratings were strongly associated with both adoption and sustainment. Implementation barriers were clustered in the inner setting, individual and innovation CFIR domains, and focused on available resources, innovation recipients and innovation design constructs. The biggest challenges to feasibility were related to constraints in the overall care delivery system, including time pressure, service delivery limitations, restricted resources and competing demands.

**Conclusions:**

Analyses indicate that health departments can develop quality‐based PLS that are acceptable, appropriate, and feasible for HIV prevention agencies and staff. This research is a case study for academic‐practice IS partnership that can identify actionable barriers and facilitators to adoption and sustainment in real time.

## 1 Introduction

In 2021, New York City Health Department (NYCHD) became one of the first United States (U.S.) jurisdictions to adopt a quality‐based financing (QBF) approach to HIV prevention programming, shifting away from the traditional fee‐for‐service model. In response to community feedback highlighting significant barriers in accessing quality HIV services, including stigma, lack of affirming client‐centred care and a need for more accountable health systems [1], NYCHD rolled out a QBF model in their PlaySure Network 2.0 (PSN 2.0) funding cycle, which provides HIV prevention contracts to agencies across the city.

The majority of research on performance‐based financing (PBF) for HIV has been conducted in sub‐Saharan Africa, with mixed results [2]. Scoping reviews reveal a lack of coherent theoretical justification or conceptual frameworks underpinning PBF designs [3], and most projects pay agencies or health systems for performance outcomes without specifying *how* these performance outcomes are to be achieved [4, 5]. More recent experimental studies that emphasize process quality, service utilization and client/provider satisfaction indicate that implementation of PBF can lead to significant health systems improvement [6].

Implementation science (IS) is the scientific study of strategies that promote the uptake of evidence‐based approaches into routine practice [7]. The PSN 2.0 funding contracts defined “quality” in terms of three programme‐level implementation strategies (PLS) described below. PLS include protocol changes or care policies that are enacted by actors within programmes (e.g. organizational leadership, programme directors, direct‐service providers) to change provider behaviour and increase care access, acceptability or uptake by clients [8]. Successful implementation of PLS is measured by adoption of the strategies by programme‐level staff. By focusing their QBF programme on implementation strategies in addition to service delivery outcomes, NYCHD held agencies accountable for both *what* was provided and *how* it was delivered.

First, funded agencies were required to conduct “universal screening” of all PSN 2.0 clients, including both routine HIV and sexually transmitted infection (STI) screening, as well as screening for supportive service needs (e.g. mental health, alcohol/substance use, housing, nutrition, childcare). Second, PSN 2.0 agencies were required to provide “holistic care,” expanding in‐house services and/or connecting clients to services that would address needs identified in universal screening. And third, PSN 2.0 agencies were required to adopt a sex‐positive, “risk‐free” approach to HIV counselling by using the GOALS Approach to Sexual History and Health (GOALS) [9]. These PLS were supported by parallel systems‐level implementation strategies, including training requirements, technical assistance (TA), data and reporting requirements, and indicator‐based accountability structures (i.e. the linkage of programme‐level implementation with funding). In Years 1 and 2 of the programme, agencies established individualized benchmarks for each quality indicator. In Year 3, 15% of agency funding was conditioned on demonstrating increases in at least 50% of these quality indicators, and agencies could receive bonus funding up to an additional 15% for increasing a greater number of indicators.

As part of its innovative approach, NYCHD collaborated with an academic research partner to conduct an IS study focused on implementation outcomes and barriers at each stage of the process. This study, called Project Partnership to Increase Access, Client‐Centered Care, and Equity in HIV Services (PACE), was fully embedded into NYCHD implementation operations, enabling three types of data sharing. First, the PACE research team received real‐time data on both programme‐ and systems‐level implementation directly from NYCHD data collection systems (e.g. training logs, meeting minutes, indicator data). Second, the research team observed learning collaboratives and other implementation sessions with funded agencies and met regularly with NYCHD implementers to discuss PSN 2.0 progress. Third, the research team conducted independent qualitative interviews and quantitative surveys with agency leadership and staff. Project PACE was informed by the Consolidated Framework for Implementation Research (CFIR), a commonly used framework in IS to identify barriers and facilitators to effective implementation [9]. Project PACE examined implementation barriers corresponding to key CFIR domains: innovation, inner setting and individuals [10].

This paper focuses on agencies’ perceptions of and experiences with the three PLS in the first 2 years of PSN 2.0, while agencies were establishing the benchmarks to use for the QBF funding structure in Year 3. We present a mixed methods examination of staff‐level implementation outcomes, focusing on acceptability, feasibility and appropriateness, as well as their association with reported adoption and sustainment. In addition, we present data on the barriers identified by agencies, mapped to CFIR domains and constructs. This study is among the first, to our knowledge, to explore utilization of PLS to promote quality HIV prevention delivery in a U.S. context.

## 2 Methods

### 2.1 Study Setting and Approach

Participants were staff at the 19 agencies funded under PSN 2.0, representing all five NYC boroughs (2 in the Bronx, 4 in Brooklyn, 10 in Manhattan, 1 in Queens and 2 on Staten Island). Agencies varied by size, with 42% (*n* = 8) serving fewer than 1000 clients per year and 32% (*n* = 6) serving over 5000 clients per year. Agencies were designated as either clinical (providing medical services in addition to HIV testing; *n* = 9) or non‐clinical (providing HIV testing, outreach and supportive services; *n* = 10). Only staff who were named on their respective PSN 2.0 grant were recruited to participate via email.

Mixed methods data collection is considered an optimal strategy for providing in‐depth understanding of implementation dynamics in large‐scale studies [11]. Quantitative surveys included standardized measures of implementation outcomes (e.g. acceptability, feasibility), and qualitative interviews added additional context to quantitative findings and focused on CFIR constructs and barriers/facilitators to implementation. Coded TA data provided the NYCHD perspective and triangulated responses from agency staff.

### 2.2 Quantitative Surveys

Surveys were distributed to 128 staff at the 19 agencies in September 2024. Sixty‐nine completed surveys (54%), averaging 3.63 staff per agency (range: 1–8), including direct service staff (*n* = 41, 59%) and supervisors (*n* = 28, 41%). Participants received a $50 gift card.

#### 2.2.1 Quantitative Measures

We measured implementation outcomes for two PLS: universal screening and GOALS. Due to concerns about survey length, direct service staff were presented with all measures for both strategies, but supervisors were presented with measures only about GOALS. Acceptability, appropriateness and feasibility were measured using the Acceptability of Intervention Measure (AIM), Intervention Appropriateness Measure (IAM) and Feasibility Intervention Measure (FIM), each of which uses a 5‐point ordinal scale ranging from “completely disagree” to “completely agree” [12]. Other measures were created for this study (see Appendix A). Cost was operationalized as the burden of each implementation strategy for staff and clients [13] and was measured using three items per strategy (e.g. “universal screening is burdensome for our clients”).

Adoption (initial/current use) and sustainment (long‐term use) were operationalized as staff reports of how often they use each strategy currently (personal adoption) and their anticipated use after PSN 2.0 ends (personal sustainment). Respondents were also asked about adoption and sustainment among other staff at their agency (general adoption and sustainment). Composite adoption/sustainment metrics were created and dichotomized as “high” versus “low.” High personal adoption/sustainment was defined as staff reporting that they used the strategy in >75% of client visits *and* would use the strategy “all or most of the time” in the future. General adoption/sustainment was coded as “high” when the respondent said that >75% of other staff at their agency currently use the strategy, and >75% of other staff would use the strategy in the future.

Finally, we measured two additional implementation constructs: leadership buy‐in and relative advantage. Leadership buy‐in was measured with a single item for universal screening and two items for GOALS; for example “agency leadership is supportive of our using the GOALS approach.” Responses were measured on a 5‐point ordinal scale, ranging from “completely disagree” to “completely agree.” Relative advantage was measured by asking participants to compare the novel implementation strategy to a traditional approach (GOALS was compared to “traditional risk assessment” and universal screening was compared with “less frequent screening”). Participants were presented with a series of items (e.g. “empowering clients,” “identifying clients’ needs”) and were asked to indicate whether the traditional strategy was better than or equal to the novel.

#### 2.2.2 Quantitative Data Analysis

We ran descriptive statistics for all measures, stratified by implementation strategy and staff type (direct service vs. supervisors). We conducted logistic regressions to assess the association between adoption/sustainment and other implementation outcomes. Due to high collinearity among AIM/IAM/FIM scores, multivariable regression with the staff perception scale variables was not appropriate [14], but we report univariable logistic regression results for each scale for universal screening adoption/sustainment, inducing odds ratios, confidence intervals, *p*‐values, *C*‐statistics (which measure discriminatory power in predicting the outcome) and Akaike information criterion (which reflects overall model fit). For GOALS adoption/sustainment, we also included staff type as a predictor in each model, since descriptive statistics suggested differences in staff perceptions by staff role, and the GOALS items were not limited by staff type. We then ran a dominance analysis to estimate the relative contributions to model predictive power for the AIM, IAM and FIM scales. Dominance analysis estimates predictors’ relative importance within a model by calculating average contribution to model fit across all possible predictor combination subset models [15]. In dominance analysis with a binary outcome, model fit is quantified using the Nagelkerke pseudo‐*R* ^2^ [16], and percentages reflect each predictor's contribution to the model's predicted variance (pseudo‐*R* ^2^), which is to be differentiated from literal “variance explained” in linear regression [15].

### 2.3 Techanical Assistance Data

Under a Data Use Agreement (DUA) with NYCHD, the research team reviewed meeting minutes from monthly TA calls with agency leads. Meeting minutes included an overview of questions and discussion about programme requirements and agency implementation. Data analysed in this paper include meeting minutes for the first 2 years of the programme (document *n* = 771).

#### 2.3.1 Technical Assistance Data Analysis

Data were extracted into REDCap [17, 18] and inductively coded to identify instances of: (a) implementation barriers; (b) implementation successes; and (c) types of TA provided. This analysis focused on codes identified as implementation barriers, classified based on its relationship to the three PLS. Barriers were thematically coded and mapped onto CFIR constructs. Instances of barrier themes by PLS were compiled into a table to examine thematic patterns within and across strategies.

### 2.4 Qualitative Interviews

Qualitative interviews (*n* = 29) were conducted virtually with participants from six agencies and included 10 (34%) supervisory staff/project leads and 19 (66%) direct service staff. Agencies were selected at random for semi‐structured qualitative interviews and included: (a) three clinical agencies (one major medical centre [MMC], one community hospital [CH] and one community health centre [CHC]) and (b) three non‐clinical agencies (two harm reduction‐focused community‐based organizations [HR‐CBOs] and one youth‐focused [Y‐CBO]). Interviews were conducted from May 2023 to March 2024 by a member of the study team. Questions focused on the staff's understanding and attitudes towards the programme, their experience with the rollout and implementation of PLS, and the impact of the programme on their agency (see Appendix B). Interviews lasted approximately 60 min, and participants received a $100 gift card. All interviews were digitally recorded and transcribed for analysis.

#### 2.4.1 Qualitative Data Analysis

We employed the rapid qualitative analysis process developed by Hamilton and Finley [19], which uses summary templates that focus on specific domains of interest. Summaries and supporting quotations are pasted into a matrix (see Appendix C), facilitating subsequent systematic identification of trends in responses. Data were coded by two members of the study team and were subsequently combined by a third team member into themes within and across PLS.

### 2.5 Ethical Approvals

This study was approved by the City University of New York Institutional Review Board (Protocol Number 2021–2119). All participants provided informed consent before participation. Data transfer from the NYC DOHMH (Department of Health and Mental Hygiene) was covered under a DUA.

## 3 Results

### 3.1 Quantitative Ratings of Programme‐Level Strategies

Table 1 presents staff scores on implementation outcomes by each PLS. Staff perceptions of acceptability (mean: 4.01 vs. 4.30), appropriateness (mean: 4.01 vs. 4.44) and feasibility (mean: 3.92 vs. 4.32) were high for both universal screening and GOALS, though they appeared slightly higher for GOALS. Mean scores for cost/burden for universal screening and GOALS were just below the midpoint for both direct service staff and supervisors, with supervisor ratings of cost for GOALS being lowest (2.52). Ratings of leadership buy‐in were also high, with supervisors rating leadership buy‐in for GOALS higher than direct service staff. Personal rates of adoption (66%) and sustainment (61%) were highest for direct service staff implementing GOALS. Both direct service and supervisory staff rated general GOALS adoption and sustainment lower than personal. Rates of personal and general adoption were comparable for universal screening, with 41%–43% reporting universal screening in more than 75% of client encounters. Predictions for high general sustainment were similar (43%), whereas only 32% of staff predicted high personal sustainment of universal screening.

**TABLE 1:** Descriptive statistics and frequencies of PSN 2.0 agency staff scores on implementation outcomes by programme‐level strategy, 2024^a^.

	Direct service staff								Supervisors				All respondents			
	Universal screening (*n* = 37)				GOALS (*n* = 41)				GOALS (*n* = 28)				GOALS (*n* = 69)			
	M	Mdn		SD	M	Mdn		SD	M	Mdn		SD	M	Mdn		SD
Acceptability	4.01	4		0.78	4.3	4.33		0.6	4.23	4.33		0.76	4.27	4.33		0.67
Appropriateness	4.01	4		0.7	4.44	4.33		0.59	4.5	4.83		0.58	4.46	4.67		0.58
Feasibility	3.92	4		0.79	4.32	4.33		0.56	4.46	4.33		0.52	4.38	4.33		0.54
Leadership buy‐in	3.95	4		0.82	4.18	4		0.71	4.28	4		0.67	4.22	4		0.69
Cost^b^	2.95	3		0.88	2.76	2.33		1.03	2.52	2.67		0.96	2.66	2.33		1

	High		Low		High		Low		High		Low		High		Low	
	*N*	%	*N*	%	*N*	%	*N*	%	*N*	%	*N*	%	*N*	%	*N*	%
Adoption: Personal	15	41%	22	59%	27	66%	14	34%	8	31%	18	69%	35	52%	32	48%
Adoption: General	16	43%	21	57%	18	44%	23	56%	5	19%	22	81%	23	34%	45	66%
Sustainment: Personal	12	32%	25	68%	25	61%	16	39%	12	46%	14	54%	37	55%	30	45%
Sustainment: General	16	43%	21	57%	17	41%	24	59%	5	19%	22	81%	22	32%	46	68%
Adoption/sustainment: Personal^c^	11	30%	26	70%	21	51%	20	49%	8	31%	18	69%	29	43%	38	57%
Adoption/sustainment: General^d^	13	35%	24	65%	16	39%	25	61%	3	11%	24	89%	19	28%	49	72%

Abbreviations: M, mean; Mdn, median; SD, standard deviation.

^a^ GOALS questions were shown to direct service staff (*n* = 41) and supervisors (*n* = 28). Universal screening questions were shown only to direct service staff. Questions on universal screening and GOALS are based on a 1−5 Likert scale (completely disagree to completely agree).

^b^ Cost is operationalized as perceived staff burden associated with the programme‐level strategy.

^c^ High personal adoption/sustainment pertains to staff who indicated *both* high personal adoption *and* high personal sustainment.

^d^ High general adoption/sustainment (adoption and sustainment among other staff at their agency) pertains to staff who indicated *both* high general adoption *and* high general sustainment.

Relative advantage ratings are presented in Figure 1, stratified by strategy and sorted by staff's preference for the new strategy. GOALS was overwhelmingly preferred to traditional risk assessment regarding reducing stigma (96%), using language that makes clients feel comfortable (93%) and empowering clients (88%). Universal screening was also largely preferred to less frequent screening for identifying clients’ needs (89%) and educating clients about services available to them (84%). Only 51% of respondents said that universal screening was preferred for reducing the burden on clients.

**FIGURE 1:**
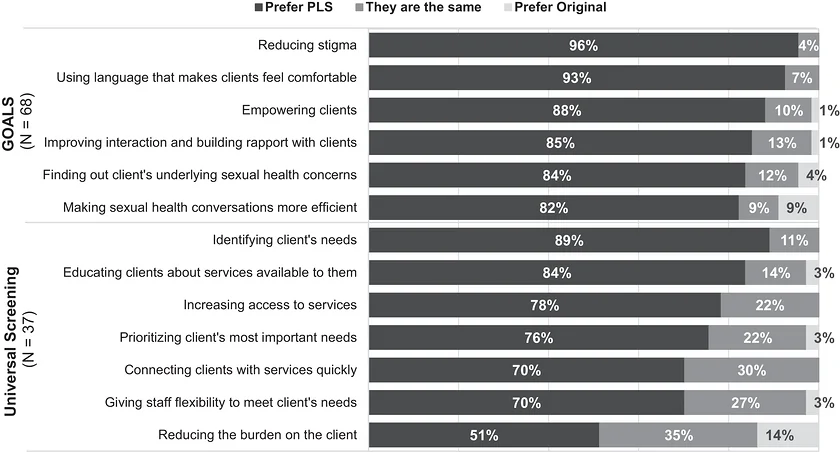
PSN 2.0 agency staff assessment of relative advantage of programme‐level strategy (PLS) compared to original approach, stratified by PLS, 2024.

### 3.2 Association Between Strategy Perceptions and Adoption/Sustainment

The results of the regressions examining associations between staff perceptions and reported adoption/sustainment of each PLS are presented in Table 2. For GOALS, after adjusting for staff type, personal adoption/sustainment was associated with acceptability, appropriateness, feasibility and leadership buy‐in, while general adoption/sustainment was associated with feasibility and leadership buy‐in. For universal screening, acceptability, feasibility and appropriateness were associated with both personal and general adoption/sustainment, but leadership buy‐in was associated only with personal (Table 2). Notably, cost was not associated with any of the adoption/sustainment outcomes.

**TABLE 2:** Univariable exploratory regression models for associations between PSN 2.0 agency staff perceptions and reported programme‐level strategy (PLS) adoption and sustainment, 2024.

	GOALS outcomes (*n* = 68)						Universal screening outcomes (*n* = 37)			
	Staff perception		Staff role				Staff perception			
	OR [95% CI]	*p*	OR [95% CI]	*p*	*c*	AIC	OR [95% CI]	*p*	*c*	AIC
**High personal adoption and sustainment**										
Acceptability	3.7 [1.4−10]	**0.010**	2.5 [0.8−7.5]	0.109	0.70	86.6	48.7 [4.2−562.9]	**0.002**	0.89	30.0
Appropriateness	3.5 [1.3−9.6]	**0.016**	2.8 [0.9−8.4]	0.068†	0.70	88.1	14.2 [2.1−96]	**0.007**	0.83	36.7
Feasibility	3.3 [1.1−9.3]	**0.028**	3.1 [1.01−9.3]	**0.049**	0.68	89.4	20.1 [2.4−167.1]	**0.005**	0.86	34.2
Leadership buy‐in	2.3 [1.04−5.1]	**0.039**	2.7 [0.9−8.1]	0.069†	0.70	90.2	4.9 [1.5−15.6]	**0.008**	0.79	39.6
Cost	0.71 [0.4−1.2]	0.196	2.6 [0.9−7.6]	0.075†	0.64	93.2	0.82 [0.4−1.9]	0.628	0.57	48.8
**High general adoption and sustainment**										
Acceptability	2.5 [0.9−7.1]	0.094†	5.4 [1.4−21.7]	**0.017**	0.74	76.5	5.8 [1.3−25.6]	**0.020**	0.75	44.0
Appropriateness	2.8 [0.9−8.9]	0.074†	6 [1.5−24.2]	**0.012**	0.01	76.0	7.1 [1.5−33.7]	**0.013**	0.77	42.8
Feasibility	5.7 [1.5−21.3]	**0.010**	7.9 [1.8−34.2]	**0.006**	0.77	71.5	13 [2−85.3]	**0.008**	0.85	38.6
Leadership buy‐in	3.5 [1.3−9.6]	**0.016**	6.6 [1.6−27.8]	**0.010**	0.77	72.5	2.5 [1−6.3]	0.052†	0.69	47.8
Cost	0.95 [0.5−1.7]	0.854	5.2 [1.3−20.3]	**0.018**	0.67	79.6	1.2 [0.6−2.7]	0.620	0.53	51.7

*Notes*: Table includes exploratory regression models predicting high personal adoption/sustainment and high general adoption/sustainment for GOALS and universal screening. Staff perception scale predictors include acceptability, appropriateness, feasibility, leadership buy‐in and cost. Each scale is used as a predictor in a separate model, due to collinearity among the AIM/IAM/FIM scales. All GOALS outcome models also include staff role as a predictor (direct service staff vs. supervisors). Universal screening questions were shown only to direct service staff. High personal adoption/sustainment means that the respondent reported that they use the PLS in >75% of their client visits, and that in the future, they will use it most/all of the time. High general adoption/sustainment means that the respondent reported that >75% of staff use the PLS and >75% of staff will use the PLS in the future. Bolding indicates *p* < 0.05; † indicates *p* < 0.10. *N* = 67 for models with GOALS personal adoption/sustainment.

Abbreviations: AIC, Akaike information criterion; AIM, Acceptability of Intervention Measure; CI, confidence interval; FIM, Feasibility Intervention Measure; IAM, Intervention Appropriateness Measure; OR, odds ratio.

Figure 2 presents dominance analysis results for all four outcomes. All three predictors shared some degree of importance, with dominance analysis results ranging from 16% to 65% per outcome. The higher the percentage, the more the predictor (staff perceptions) contributes to the model's predictive power for that outcome (adoption/sustainment of each PLS). For personal adoption/sustainment, acceptability demonstrated the highest relative importance for both universal screening (56%) and GOALS (60%). For universal screening, feasibility demonstrated higher relative importance than appropriateness (28% vs. 16%), but the opposite pattern emerged for GOALS (14% vs. 26%). For general adoption/sustainment, feasibility was the most important predictor for both universal screening (62%) and GOALS (65%).

**FIGURE 2:**
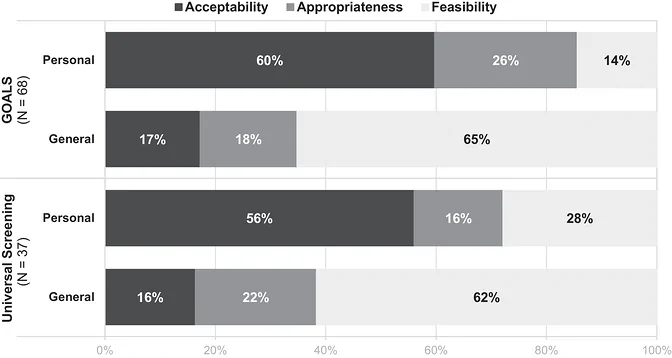
Dominance analysis results among PSN 2.0 agency staff for predictors of adoption and sustainment, stratified by programme‐level strategy, 2024. The higher the percentage, the more the predictor (staff perceptions of acceptability, appropriateness and feasibility) contributes to the model's predictive power for each outcome (personal or general adoption/sustainment of universal screening and GOALS).

### 3.3 Qualitative Data on Acceptability, Feasibility and Appropriateness

To better understand factors influencing adoption and sustainment, we focused on qualitative findings related to acceptability, feasibility and appropriateness for each PLS.

#### 3.3.1 Universal Screening

Participants discussed several benefits of universal screening associated with acceptability and appropriateness. First, universal screening was credited with increasing the efficiency of the intake process, allowing staff to better coordinate care. This efficiency was described as a direct benefit to the clients, for both the speed with which they received services and the reduced potential trauma inherent in needing to explain the same issues and needs to multiple people during a visit.

*So you have a central system that conducts the assessment and the intake and then refers them within the agency for services and also out in the community for additional services. So that is great because you don't want to re‐traumatize people having to tell their story again and again*. (Agency Leadership, HR‐CBO)

Another central theme was the extent to which universal screening increased practitioners’ ability to identify client needs that they had not fully assessed in the past.

*There are things in the PlaySure [PSN 2.0] intake that were not asked before, specifically naming the different resources such as housing, food, childcare, hygiene, and even adding extra categories. So I think that it's broadened our view in that way*. (Outreach Staff, CH)

Respondents also identified several factors that limited the feasibility of universal screening. The sheer burden of the screening volume, especially as a new requirement, was daunting: “*It [universal screening] had to be on every single patient…that's like 3,000 people here, but we're getting there”* (Medical Provider, CHC).

Universal screening was described as less feasible in outreach settings or other components of service delivery that were supposed to be more streamlined.

*The screener is so long and it's required of everybody… it's just not that feasible to collect all this data on people who are maybe out at a party*. (Outreach Staff, MMC)

This tension between the value of comprehensive assessment and the time constraints of client encounters was experienced in clinical settings as well:

*I think intakes are longer now because of PlaySure, unfortunately…sometimes that does honestly mess up our clinic workflow a little because now the doctors are like, “Hey,” and I'm like, “Oh, I'm trying to finish these questions and they're really important questions.” But also, you don't want it to feel rushed. You don't want the patient to feel like you are ignoring them or you're just trying to get through the laundry list…But I also think that's just the nature of being in a clinical setting, unfortunately, you only have so much time*. (Outreach Staff, CH)

#### 3.3.2 Holistic Care

Respondents overwhelmingly appreciated that the focus on holistic care enabled them to pay for critical services that had not been included in prior HIV prevention contracts. Mental health was the top‐cited advantage for this PLS, especially for agencies that were already providing clinical care:

*I'd say it's been great to have an addition for a mental health provider in [our neighborhood]. So, that has historically been one of the larger barriers for all of our participants. And that's been a huge, huge, huge addition that's come with PlaySure*. (Programme Manager, HR‐CBO)

Other service innovations attributed to this strategy included care navigation, rapid STI testing and telehealth. This PLS was also credited with helping agencies enhance services to meet client needs, including extended hours, same‐day visits and transportation assistance. This strategy gave the agencies flexibility, because services that agencies always considered central to engagement in HIV prevention were now explicitly recognized by their funding contract as such.

*In the past, if somebody came in for HIV and STI testing and maybe had something else going on that they wanted help around, that would be a harder thing to cover. But now we can. We are like, ‘This is our comprehensive package of visits’*. (Programme Manager, MMC)

Participants also noted that providing holistic care was not always feasible in their settings, and expressed feeling challenged by having to be all things to all clients:

*I personally find myself overwhelmed sometimes with the amount of different things that we can help them with, because it's like I can't be a specialist in all of these areas, and I don't know all of these things*. (Navigator, Y‐CBO)

#### 3.3.3 GOALS

Acceptability and appropriateness for GOALS centred around the strategy's impact on reducing stigma and improving communication with clients. The strategy was described as “*not putting judgement on the client*” (Navigator, Y‐CBO), “*being more sex positive*” (Programme Coordinator, MMC), “*building rapport*” (Navigator, Y‐CBO) and “*destigmatizing things*” (Mental Health Counsellor, HR‐CBO). This change in interaction was specifically credited with increasing the number of clients who accepted HIV testing.

The way in which GOALS centres the client experience was also described as a significant advantage for both clients and providers.

*[GOALS] makes these assessments for risk much more based on what are the priorities and interests of the client…so, having this be a way of putting like an official stamp on the idea of treating people like human beings when you ask them about health‐related practices, it's great. Huge fan*. (Mental Health Counsellor, HR‐CBO)

Challenges to GOALS centred around the difficulty in changing established patterns of risk assessment and documentation, especially within larger institutions.

*I feel like when we're used to taking history a certain way, changing that is going to take some time. And so, there's been a lot of misses on my side in terms of asking open‐ended questions…because the way that our EMR is structured right now, it's still the old way, so I have to over step it, or still use it*. (Clinician, CHC)

### 3.4 Reported Barriers to Program Level Strategies

A total of 129 mentions of specific barriers to PLS were identified in TA meeting minutes and grouped into 11 barriers corresponding to three key CFIR domains: inner setting, individuals and innovation (Table 3). Inner setting barriers accounted for 41% of codes across PLS, with staffing shortages emerging as the most frequently coded barrier in this domain (17% of codes). Barriers related to individuals accounted for 22% of codes, with innovation recipients (i.e. client reactions to the PLS) accounting for 16% of codes. Innovation barriers accounted for 37% of codes, with the plurality (20%) focused on confusion or lack of clarity about PLS requirements.

**TABLE 3:** Frequencies of programme‐level strategy (PLS) barriers among PSN 2.0 agency staff by domain, barrier and PLS, 2023–2024.

			Programme‐level strategies							
			Universal screening		Holistic care provision		GOALS		Total (across strategies)	
CFIR domain	CFIR construct	Barriers	*N*	%^a^	*N*	%^a^	*N*	%^a^	*N*	%^a^
Inner setting	Available resources	Volume/capacity burden	3	6%	6	11%	1	4%	10	8%
		Documentation burden	5	10%	3	5%	2	8%	10	8%
		Process burden	0	0%	2	4%	1	4%	3	2%
		Resource burden	0	0%	8	15%	0	0%	8	6%
		Staffing shortage	5	10%	16	29%	1	4%	22	17%
		**Total**	**13**	**26%**	**35**	**64%**	**5**	**20%**	**53**	**41%**
Individuals	High‐level leaders	Lack of leadership buy‐in	2	4%	0	0%	5	21%	7	5%
	Innovation deliverers	Provider discomfort	1	2%	0	0%	0	0%	1	1%
	Innovation recipients	Client reactions	12	24%	6	11%	2	8%	20	16%
		**Total**	**15**	**30%**	**6**	**11%**	**7**	**29%**	**28**	**22%**
Innovation	Innovation design	Lack of clarity/confusion	15	30%	6	11%	5	21%	26	20%
		Materials or systems not ready	4	8%	6	11%	3	13%	13	10%
	Relative advantage	Not feasible, useful or necessary	3	6%	2	4%	4	17%	9	7%
		**Total**	**22**	**44%**	**14**	**26%**	**12**	**51%**	**48**	**37%**
		**Total (across barriers)**	**50**	**39%** ^b^	**55**	**43%** ^b^	**24**	**18%** ^b^	**129**	**100%**

^a^ Column percentages. For example, within universal screening, volume/capacity burden accounted for 6% of barriers mentioned (*n* = 3/50), and inner setting barriers, as a whole, accounted for 26% of barriers mentioned (*n* = 13/50). Across all programme‐level strategies, inner setting barriers accounted for 41% of barriers mentioned (*n* = 53/129), and volume/capacity burden accounted for 8% (*n* = 10/129).

^b^ Row percentages. For example, 39% of barriers mentioned across all technical assistance calls (*n* = 50/129) pertained to universal screening.

*Source*: Technical assistance meeting minutes with PSN 2.0 agency staff.

Across the three PLS, barriers were more commonly focused on holistic care provision (43%) and universal screening (39%), compared to GOALS (18%). Within each PLS, slightly different barriers emerged as most frequent. Concerns about client reactions were the most common barrier expressed for universal screening: “[Staff] *have received a lot of denials for screenings. [It takes] 15–20 min [to] screen. As part of the intake—some people don't want to do it. People just want their meds or STI test”* (MMC). Staffing shortages and resource burden were most common for holistic care provision: “*Due to lack of staffing, there has been a reduction of services*” (CBO); “*They are limited in the number of participants they can have in the space at once*” (CHC). Lack of leadership buy‐in was one of the most common barriers to GOALS implementation: “*Support from management is necessary*” (CHC).

### 3.5 Qualitative Analysis of CFIR Domains

In the inner setting domain, multiple participants reported on the lack of available resources, recapitulating the challenges with both resource burden and staffing shortages.

*The direct service staff were feeling super, super spread thin…and they were very overwhelmed by the massive expectations of them doing so much screening and so much mandatory education, even when clients weren't necessarily wanting that*. (Programme Coordinator, HR‐CBO)

In the innovation domain, confusion about universal screening was related to the way that agencies were asked to document this strategy. During screening, agencies were asked to document whether a client was “interested” in a particular service, and then whether all interested clients had been linked to services. But the definition of “interested” was challenging for agencies, and they were concerned that this method of documentation would make it appear that they were not providing sufficient care to their clients.

*I think being interested in something and wanting something is two different things, and they [NYCHD] look at that as if you check interested, that means that the person should have been linked to that service, but that's not how I look at it. If somebody's interested, they're just inquiring stuff about what's going on. So I think they need to change the format of that question…That's the biggest challenge*. (Navigator, HR‐CBO)

The other critical innovation design barrier involved delays in the availability of materials and systems at the start of implementation. Because the NYCHD was newly creating the systems‐level resources that supported PLS, some supports were not in place at the start of implementation. This delay created significant stress for agencies: *“The screener was just released…six months after the rollout. It's like we had to work with and invent something from nothing, which in the beginning was a big struggle internally for us”* (Clinician, CHC). Multiple agencies expressed tension with NYCHD around requirements, and expressed concerns that they were being held accountable for failing to complete tasks when the systems to report on those tasks were not available: “*Certain [reporting tools] weren't working. Because it's new, which is okay. But also, you can't be mad at us, the people that's working. We're trying to do everything that we can”* (Navigator, Y‐CBO).

## 4 Discussion

This analysis demonstrates that health departments can develop quality‐based PLS that are acceptable, appropriate and feasible for HIV prevention agencies and their staff. Staff attitudes towards each of the strategies are consistent with previous studies about barriers to and facilitators of universal screening, integrated care services and opt‐out testing, but with some notable differences. In past research, providers acknowledge the importance of screening clients for social determinants of health, but few report actually assessing them, citing lack of training, time constraints and insufficient resources to address identified needs [20]. Each of these barriers was present in our data, but most participants also reported overcoming them. As such, universal screening was described as efficient and effective in identifying clients’ needs and was lauded as expanding both staff and clients’ understanding of available services.

Past research on holistic care or “wrap‐around” services have been elevated as key to effective programme implementation [21] and sustained engagement [22], but staff report that it can conflict with low‐threshold service delivery and requires additional staffing [23]. In our study, participants reported that a focus on holistic care allowed agencies to expand delivery of critical services and provided flexibility to serve more clients. The service components most frequently mentioned by participants under holistic care were mental health, STI testing and telehealth. Our data highlight the importance of health departments’ provision of meaningful TA to help agencies maintain flexibility with documentation requirements, support client‐centred care and build strong referral pathways for supportive services. Findings suggest that collaborations should extend beyond standard “memoranda of understanding” (MOU) relationships and foster genuine partnerships with agencies that specialize in support services to fill gaps and decrease the burden on agency staff.

Financial support of staff and other resources for these services was considered “game‐changing” for agencies in meeting their clients’ needs. GOALS risk‐free HIV prevention counselling scored highest on quantitative acceptability and feasibility measures, and over 80% of staff preferred GOALS to traditional risk screening on each dimension of the relative advantage scale. Qualitative interviews reinforced these survey data and underscored staff beliefs that GOALS reduced stigma, centred client experience and increased rapport. These findings are consistent with past research citing stigma as a critical barrier to routine HIV testing [24, 25, 26]. In each case, staff acceptability ratings were directly related to perceptions of the strategy's positive impact on their clients’ experience of care. These data suggest that implementation innovations to improve quality HIV prevention services may be welcomed by front‐line implementers.

However, adoption of these PLS was not yet universal. Two‐thirds of direct service staff reported high adoption of GOALS, but only 41% reported high adoption for universal screening. For GOALS, participants reported their own adoption and sustainment as higher than that of other staff, but for universal screening, participants rated their own adoption and sustainment lower than other staff. For both strategies, *acceptability* emerged as the biggest driver of *personal* adoption and sustainment, but *feasibility* emerged as the biggest driver of staff's perceptions of adoption and sustainment among *other* staff at their agency. These findings align with past research on the importance of skill‐building, tangible resources, and easy integration into existing systems as facilitators of HIV prevention practice change [22, 27].

In our analysis of CFIR barriers, implementation of PLS was hindered by the lack of available resources in the inner setting: staffing shortages, limited funding, overburdened programmes and competing demands to approach care differently. Across each PLS, participants described a tension between the quality of care they would like to provide and a system that was not set up to provide that care. These data suggest three lessons for future QBF initiatives. First, system‐level actors (such as NYCHD) who are using QBF may consider providing additional supports that directly address feasibility barriers. These supports may be more effective if fully developed and in place before any programme‐level implementation begins. Second, quality‐focused PLS that are clearly connected to client‐level outcomes and integrated into existing workflows may prevent staff from feeling that these new responsibilities are taking away from existing client care. And third, QBF initiatives must recognize that agencies are funded by other contracts whose requirements might run counter to a QBF model. More work is needed on how a QBF approach might be integrated into a care context focused almost exclusively on billable services and reimbursements.

These findings also highlight the extent to which the NYCHD emphasis on PLS as quality indicators has not been widely used or studied [2]. Large‐scale QBF initiatives in the United States change reimbursement structures but fail to specify the *strategies* that agencies might use to improve outcomes and meet new reimbursement standards [28]. Research comparing trajectories of performance in response to QBF initiatives focus on organizational characteristics (hospital type, bed size, urban/rural) and cite the need to better understand the *processes* by which institutions might improve care [29]. More work is needed on the integration of PLS into QBF models, and the mechanisms through which changing care delivery strategies can improve outcomes.

Our data are subject to several limitations. This was an analysis of a single health department's QBF strategy and was conducted in collaboration with the implementation process, not as a research trial. These data focus on the first 2 years of implementation, before QBF outcome accountability went into effect. Future research will focus on the impact of the NYCHD strategy on outcomes data. The interpretation of regression findings are limited by the small sample size. These data are restricted to self‐reported implementation outcomes and perceptions of staff. Future analyses in this project will include examination of client experience data and analyses of programme‐level changes in service delivery tied to implementation outcomes.

## 5 Conclusions

As funding shifts require jurisdictions and programmes to do more with less, focusing on the quality of HIV prevention services is paramount. Systems‐level actors may consider developing and incentivizing quality‐focused implementation strategies that focus on what is delivered and how it is delivered. Data suggest that adoption and sustainment of these strategies rely both on perceived acceptability and impact for clients and on the feasibility of strategy integration into a larger service delivery infrastructure. This research also highlights the potential of academic‐practice IS partnership to identify actionable barriers to and facilitators of adoption and sustainment *in real time*. Future work should use similar models to support the use of innovative PLS strategies to effect positive change in our HIV prevention and care system.

## Author Contributions

SAG and AMDR conceptualized this analysis. SAG, AMDR and IKH performed study interviews; AMDR and IKH conducted qualitative coding; AMDR, IKH and SAG created TA coding frameworks, and conducted qualitative coding/analysis. SAG and IKH created the quantitative survey; LS performed quantitative analyses. SAG, LS and AMDR wrote the paper. All authors revised the manuscript for content and have read and approved the final manuscript.

## Funding

Funding for this project was provided by the Department of Health and Human Services, Centers for Disease Control and Prevention (U01PS005239; S.A. Golub, PI).

## Conflicts of Interest

The authors declare that they have no conflicts of interest or competing interests.

## Disclaimer

The findings and conclusions in this report are those of the authors and do not necessarily represent the official position of the Centers for Disease Control and Prevention.

## Supporting information




**Appendix A**: Custom‐created quantitative data collection measures: Cost (Burden), Leadership Buy‐In, Adoption, and Sustainment.
**Appendix B**: Interview Guides for Program Lead/Supervisors and Direct Care Staff.
**Appendix C**: Summary Template for Rapid Qualitative Analysis of Qualitative Interviews with Program Lead/Supervisors and Direct Care Staff.

## Data Availability

The data that support the findings of this study are available on reasonable request from the corresponding author. The data are not publicly available due to privacy or ethical restrictions.
