# Supplementary material for: Implementation Outcomes and Lessons From a Health‐Department‐Driven Quality‐Based Financing Approach to HIV Prevention Programmes in New York City, United States: A Mixed Methods Analysis
Source: J Int AIDS Soc. 2026 Oct 8;29(Suppl 5):e70194. doi: 10.1002/jia2.70194 (PMC13650413; doi:10.1002/jia2.70194)
Supplement: Supplementary file 1 [file JIA2-29-e70194-s001.pdf]

Partnership to Increase Access, Client-Centered Care,
and Equity in HIV Services (CDC, U01PS005239)

Supplemental File

Implementation Outcomes and Lessons from a Health-Department-Driven Quality-Based Financing Approach to HIV Prevention Programs in New York City: A Mixed Methods Analysis

Sarit A. Golub[1,2,3 §], Ariel M. de Roche[1], Lila Starbuck[1], Imani Hall[1], Brittany C. Wilbourn[4]

1 Hunter Alliance for Research and Translation, Department of Psychology, Hunter College of the City University of New York (CUNY)
2 Einstein-Rockefeller-CUNY Center for AIDS Research, Albert Einstein College of Medicine
3 Department of Psychology, Basic and Applied Social Psychology PhD Program, Graduate Center of the City University of New York
4 HIV Research Branch, Division of HIV Prevention, Centers for Disease Control and Prevention, Atlanta, GA, USA

CONTENTS

Appendix A: Custom-created quantitative data collection measures: Cost (Burden), Leadership Buy-In, Adoption, and Sustainment 2

Appendix B: Interview Guides for Program Lead/Supervisors and Direct Care Staff 4

Appendix C: Summary Template for Rapid Qualitative Analysis* of Qualitative Interviews with Program Lead/Supervisors and Direct Care Staff 10

Appendix A: Custom-created quantitative data collection measures: Cost (Burden), Leadership Buy-In, Adoption, and Sustainment

Cost (Burden)

GOALS (α = 0.696)
- Implementing GOALS takes time away from other important work at our agency.
- GOALS Training was a burden on our staff.
- It's hard to use GOALS because of other funding that requires "risk-based" language

Universal Screening (α = 0.699)
- Universal screening is tedious/burdensome for our staff
- Universal screening takes time away from other important work we do to help our clients
- Universal screening is burdensome for clients

Response Values:
1. Completely Disagree
2. Disagree
3. Neither Agree nor Disagree
4. Agree
5. Completely Agree

Leadership Buy-In

GOALS[1] (α = 0.751)
- Agency leadership is supportive of our using the GOALS Approach
- Agency leadership recognizes the value of GOALS for service provision at our agency

Universal Screening
- Agency leadership is supportive of a move to universal screening

Response Values:
1. Not at All
2. A little bit
3. Somewhat
4. To a large extent
5. Completely

[1] One item was excluded due to low Cronbach's alpha value for the scale, prior to its exclusion: "Agency leadership was a barrier to GOALS implementation." *(reverse-coded)*

Adoption and Sustainment

Personal Adoption
- o In what percentage of your client visits do you personally use the GOALS Approach?
- o In what percentage of your client visits do you personally do universal screening?

Personal Sustainment
- o When PSN 2.0 ends, how often do you think YOU will use GOALS?
- o When PSN 2.0 ends, how often do you think YOU will do universal screening?

General Adoption
- o What percentage of staff at your agency use the GOALS approach?
- o What percentage of staff at your agency do universal screening with clients?

General Sustainment
- o When PSN 2.0 ends, how often do you think other staff at your agency will use GOALS when talking to clients?
- o When PSN 2.0 ends, what percentage of staff at your agency do you think will continue to do universal screening?

Response Values:

Percentages
1. Less than 10%
2. 10-25%
3. 26-50%
4. 51-75%
5. 76-100%

Frequency
1. Never
2. Rarely
3. Sometimes
4. Often
5. Most/All the Time

Dichotomized Outcome Values
- o High Adoption/Sustainment:
  - o "High" on both Adoption and Sustainment
  - o Value 5: Most/All of the Time, or 76-100%
- o Low Adoption/Sustainment
  - o "Low" on Adoption and/or Sustainment
  - o Values 1-4: Never to Often, or 0-75%

Appendix B: Interview Guides for Program Lead/Supervisors and Direct Care Staff

Program Lead/Supervisor Interview

Section #1: Orientation to the Agency's Program

Key Research Questions
- How does HIV prevention – and PSN 2.0 specifically – fit in to the larger services the agency provides?
- Who are the agency's PSN 2.0 clients and how are they related to the client population as a whole?

1. To start, can you give a brief self-introduction, including: your name, pronouns, and your roles and responsibilities here at [SITE NAME].
2. How has your work changed in response to the PSN 2.0 grant funding?
3. I know that PSN 2.0 is potentially just one of many contracts you have. I would like to get a better understanding of how the PSN 2.0 model and funding fits into the rest of the work of your agency.
4. I'd like to get a sense in general of the clients that you serve. How would you describe your overall client population?
5. Now can you tell me about the clients who are most likely to be served through PSN 2.0? How are these clients the same or different from your general population?
6. Tell me about any clients who are falling through the cracks of PSN 2.0 – meaning you feel that they would benefit from PSN 2.0 services, but they aren't accessing them.
7. Has PSN 2.0 made it *easier* for certain patients to access care?

Section #2: Agency's Experience with PSN 2.0

Key Research Questions [this is what you are trying to "get out" of this section]:
- How has the agency experienced the roll-out of PSN 2.0?
- How has PSN 2.0 rollout impacted their program and staff?

1. Now I'd like to talk a bit more about the nitty gritty of PSN 2.0 and its implementation. To start, what's your sense of main goal of PSN 2.0 from the perspective of the health department?
2. How well do you think the goals of PSN 2.0 fit with your own agency's model and mission?
3. Tell me a little bit about your experience of the PSN 2.0 rollout – what went smoothly and what was difficult?
4. To what extent did your agency need to develop new processes for PSN 2.0? How has that gone?
5. How was – and is – information about PSN 2.0 shared with staff at [AGENCY]? Is information shared with all staff or just staff who are named on the project?
6. What aspects of PSN 2.0 have been easiest or quickest to implement?

7. Which have been the most challenging or experienced the most delays?
8. How has PSN 2.0 impacted your staff?
9. How has PSN 2.0 impacted the relationship between staff and clients?

Section #3: Agency Culture and Environment

Key Research Questions
- What is the agency culture like?
- How does the "inner context" of the agency impact PSN 2.0 implementation?

1. One of the things that we are really interested in is how the PSN 2.0 model works at agencies with different organizational styles and cultures. How would you describe the culture at [AGENCY NAME]?
    - *Clarification, if necessary*: What is it like to work there; how do you interact with your coworkers? What are your interactions like with your clients?
2. How would you describe the values and beliefs that are most important to your organizations?
3. Do you think there are any differences in these values and beliefs among individuals with different roles in your organization?
4. How do you think these organizational values align with values and priorities held by your clients?
5. Are there any things about your agency's culture that you think has made it easier or more challenging to implement PSN 2.0?
6. How are organizational decisions made at [AGENCY NAME]?
    - *Clarification, if necessary*: Who is involved? What is the process, if there is one?
7. How, if at all, are decisions related to PSN 2.0 similar or different from other contracts / work streams?

Section #4: PSN 2.0 Impact and Mechanisms

Key Research Questions
- How has PSN 2.0 implementation impacted their clients?
- How has PSN 2.0 impacted the hypothesized "mechanisms of action" for this strategy: availability, accessibility, and equity?

1. In this last section, I'd love to talk to you about how you think PSN 2.0 is impacting (or will impact) your clients. Can you start by just telling me in general what impact you think the program has had?
2. What are some of the unexpected or inadvertent impacts the program has had?

3. Let's talk for a minute about **availability** of services. How has PSN 2.0 impacted availability of services at your site? ['availability' meaning the services you are able to provide or your ability to provide more comprehensive care]
    a. How do you think this impact will change over time?
    b. What things have or might get in the way of this impact?
    c. What would facilitate *positive* change in availability of services?
4. OK now let's talk about **accessibility**. How has PSN 2.0 impacted accessibility of services at your site? ['accessibility' meaning things like colocation of services, reducing steps or handoffs…]
    a. How do you think this impact will change over time?
    b. What things have or might get in the way of this impact?
    c. What would facilitate *positive* change in accessibility of services?
5. Finally, let's talk about **equity**.
    a. How does your agency define equity?
    b. How, if at all, has PSN 2.0 impacted your agency's definition of or thoughts around equity
    c. How has PSN 2.0 impacted equity at your site?
    d. How do you think this impact will change over time?
    e. What things have or might get in the way of this impact?
    f. What would facilitate *positive* change?

<u>Section #5: PSN 2.0 Wrap up</u>

**Key Research Questions**
• Final thoughts

1. We're almost at the end of our interview. I'm going to pause for a moment to check to see if my colleagues have any last questions for you.
2. My very last question: is there any other information that you think is important for us to know that hasn't been covered in this interview?
3. **IF TIME ALLOWS: Sentence Completions** I want to do a few fill-in-the-blanks, where I start a sentence and then you fill it in with whatever word or phrase.
    a. The biggest benefit of PSN 2.0 is …
    b. The biggest challenge of PSN 2.0 is …
    c. PSN2.0 would be a better fit with our organizational culture if …
    d. If I could change one thing about the physical space where I work to make my job easier, it would be … because …
    e. PSN2.0 makes me feel hopeful because …

Direct Care Staff Interview

Section #1: Staff Relationship to PSN 2.0

Key Research Questions

- What is this staff member's general understanding of PSN 2.0 and what are their attitudes toward the program?
- How does the staff member think that PSN 2.0 has impacted their work?

1. To start, can you give a brief self-introduction, including: your name, pronouns, and your roles and responsibilities here at [SITE NAME].
2. I know that PSN 2.0 is potentially just one part of the work you do here. Can you tell me a little bit about your work on PSN 2.0 specifically and how it fits into the rest of the work that you do?
3. Can you tell me your perspective on what the main goals of PSN 2.0 are? What is the health department trying to achieve with this program?
4. How well do you think the goals of PSN 2.0 fit with your own agency's model and mission?
5. From your perspective, what are the best parts of PSN 2.0?
6. What things about PSN 2.0 are most challenging?
7. In general, how would you say that PSN 2.0 has impacted the work that you do?

Section #2: Staff Experiences with Program Components

Key Research Questions

- What PSN 2.0 program components has the staff member been exposed to?
- What are staff's experience and opinions of PSN 2.0 program components?

Now I'd like to ask you some specific questions about the different components of the PSN 2.0 program and its requirements for agencies.

1. Let's start with **training**. What kind of training have you received as a result of the PSN 2.0 program?
    - *Did you receive any training on the GOALS approach to sexual health?*
    - *Did you receive any anti-stigma or anti-racism training?*
    - *Did you receive any training on PSN 2.0 program administration – like the QIM tool or eSHARE?*
2. How would you rate the training that you've received? What was useful and what wasn't?
3. To what extent do you think that the training provided by PSN 2.0 has changed the way in which care is delivered to clients?

4. Now let's talk about the client and staff experience surveys. Have familiar are you with the **STAFF** experience survey? Have you taken it? *[If no experience, skip to next question]*
    i. What are your thoughts about the **staff** experience survey?
    ii. What feedback or information have you received about the results from the **staff** survey and/or how they are being used at your agency?
5. Now, how familiar are you with the **CLIENT** experience survey? Have you seen it? *[If no experience, skip to next question]*
    i. What are your thoughts about the client experience survey? Have clients given you and feedback about it?
    ii. What information have you received – or will you receive – about the results of the client survey and/or how they are being used at your agency?
6. What other aspects of the PSN 2.0 model are your familiar with – do you know about other program requirements or goals?
7. [*If yes*]: What are your opinions about these requirements? How do you think they have impacted care at your agency?

Section #3: Agency Culture and Environment

Key Research Questions [this is what you are trying to "get out" of this section]:

- What is the agency culture like?
- How does the "inner context" of the agency impact PSN 2.0 implementation?

1. One of the things that we are really interested in is how the PSN 2.0 model works at agencies with different organizational styles and cultures. How would you describe the culture at [AGENCY NAME]?
    Clarification, if necessary: What is it like to work there; how do you interact with your coworkers? What are your interactions like with your clients?
2. How would you describe the values and beliefs that are most important to your organization?
3. Do you think there are any differences in these values and beliefs among individuals with **different roles** in your organization?
4. How do you think these organizational values align with values and priorities held by your **clients**?
5. Are there any things about your agency's culture that you think has made it easier or more challenging to implement PSN 2.0?
6. How was – and is – information about PSN 2.0 shared with staff at [AGENCY]?

Section #4: Changes associated with PSN 2.0

Key Research Questions

- How has PSN 2.0 impacted changes at the agency?
- How and why has this happened (or not happened)?

1. It's been about [NUMBER] months since PSN 2.0 was rolled out at your agency. In this time – since [DATE], have there been any changes to the way that HIV prevention services are organized or provided to clients?
2. I'm going to ask about some specific areas that I know that PSN 2.0 is supposed to focus on – have there been any changes in… *(NB: choose just a couple that you think are most relevant to this staff member or agency):*
    a. hiring practices or efforts to retain staff at your agency?
    b. how clients can pay for services?
    c. agency hours or accessibility?
    d. type or number of clients served?
    e. the way appointments are offered?
    f. intake forms or patient-education materials?
    g. signage in waiting rooms or bathrooms?
    h. materials on display in common areas or private areas of the site?
3. *[If any changes are reported]:* What do you think about these changes?
4. Are there changes that you think should be happening at your agency but aren't?
    - Are there gaps or blind spots in your HIV prevention program that you wish were better or easier?
    - Are there things you wish your agency would do better?
5. My very last question: is there any other information that you think is important for us to know that hasn't been covered in this interview?

Appendix C: Summary Template for Rapid Qualitative Analysis* of Qualitative Interviews with Program Lead/Supervisors and Direct Care Staff

*Adapted from A. Hamilton. *Hamilton AB, Finley EP. Qualitative methods in implementation research: An introduction. Psychiatry Res. 2019;280:112516.*

Program Lead/Supervisor Interview Summary Template

			Participant ID#:<br>Interviewed by:<br>Coded by:		
**Construct/Topic**	**Interview Question**	**Domain**	**Summary**	**Timestamp**	**Illustrative Quote**
Participant Overview	To start, can you give a brief-self introduction, including: your name, pronouns, and your roles and responsibilities.<br>How has your work changed in response to the PSN 2.0 grant funding?	staff member's job and relation to PSN 2.0			
Place of PSN 2.0 in broader agency	How does HIV prevention – and PSN 2.0 specifically – fit into the larger services the agency provides?	connection between PSN and other contracts			
	Who are the agency's PSN 2.0 clients and how are they related to the client population as a whole?	impact of PSN on clients served			
Perceptions of PSN 2.0 goals, fit, benefits and challenges	How has the agency experienced the roll-out of PSN 2.0?<br>How has PSN 2.0 rollout impacted their program and staff?	PSN 2.0 goals			
		PSN 2.0 fit with agency			
		PSN 2.0 benefits			
		PSN 2.0 challenges			

		impact on work/staff			
Agency Culture	What is the agency culture like?<br>How does the "inner context" of the agency impact PSN 2.0 implementation?	relationships among staff			
		general feel of agency			
		agency mission			
		organizational structure and decision-making			
		interaction of culture and PSN 2.0			
		other			
Impacts of PSN 2.0 Funding	How has PSN 2.0 implementation impacted their clients?<br>How has PSN 2.0 impacted the hypothesized "mechanisms of action" for this strategy: availability, accessibility, and equity?	new positions			
		expanded services			
		changes in practice			
		screener			
		data & reporting			
		availability			
		accessibility			
		equity			
		other			
Challenges of PSN 2.0 Funding	*Place things that are said into the appropriate buckets to the right…*	roll-out challenges			
		hiring challenges			
		data challenges			
		training challenges			
		culture shifts			
		practice shifts			
		burden of requirements			
		other			

Final Thoughts	Is there any other information that you think is important for us to know that hasn't been covered in this interview?	summary/additional info			
Other Observations	-	-			
Important Quotations	-	-			

Partnership to Increase **A**ccess, **C**lient-Centered Care,
and **E**quity in HIV Services (CDC, U01PS005239)

Direct Care Staff Interview Summary Template

			**Participant ID#:**<br>Interviewed by:<br>Coded by:		
**Construct/Topic**	**Interview Question**	**Domain**	**summary**	**timestamp**	**illustrative quote**
Participant Overview	Can you tell me a little bit about your work on PSN 2.0 specifically and how it fits into the rest of the work that you do?	Staff member's job and relation to PSN 2.0			
Perceptions of PSN 2.0 goals, fit, benefits and challenges	Can you tell me your perspective on what the main goals of PSN 2.0 are? What is the health department trying to achieve with this program?	PSN 2.0 goals			
	How well do you think the goals of PSN 2.0 fit with your own agency's model and mission?	PSN 2.0 fit with agency			
	From your perspective, what are the best parts of PSN 2.0?	PSN 2.0 benefits			
	What things about PSN 2.0 are most challenging ?	PSN 2.0 challenges			
	In general, how would you say that PSN 2.0 has impacted the work that you do?	impact on work			
Perceptions of the quality and impact of specific program components	What kind of training have you received as a result of the PSN 2.0 program?	training received			
	How would you rate the training that you've received? What was useful and what wasn't?	response to training			
	To what extent do you think that the training provided by PSN 2.0 has changed the way in which care is delivered to clients?	impact of training			

	Have familiar are you with the staff experience survey? Have you taken it?	experience with staff surveys			
	What are your thoughts about the staff experience survey?	response to staff surveys			
	What feedback or information have you received about the results from the staff survey and/or how they are being used at your agency?	impact of staff surveys			
	How familiar are you with the client experience survey? Have you seen it?	experience with client surveys			
	What are your thoughts about the client experience survey? Have clients given you and feedback about it?	response to client surveys			
	What information have you received – or will you receive – about the results of the client survey and/or how they are being used at your agency?	impact of client surveys			
	What other aspects of the PSN 2.0 model are your familiar with – do you know about other program requirements or goals?	experiences with other PSN 2.0 aspects			
	What are your opinions about these requirements?	response to other PSN components			
	How do you think they have impacted care at your agency?	impact of other PSN components			
Agency Culture and Environment	How would you describe the culture at your agency	agency culture			
	How would you describe the values and beliefs that are most important to your organizations?	agency values			

	Do you think there are any differences in these values and beliefs among individuals with different roles in your organization?	value differences by role			
	How do you think these organizational values align with values and priorities held by your clients?	value differences for clients			
	Are there any things about your agency's culture that you think has made it easier or more challenging to implement PSN 2.0?	agency culture and PSN 2.0			
	How was – and is – information about PSN 2.0 shared with staff at [AGENCY]?	communication about PSN 2.0			
Implementing Change at Agency Level	Have there been any changes to the way that HIV prevention services are organized or provided to clients?	PSN 2.0 changes			
	Place things that are said into the appropriate buckets to the right…	hiring practices			
		client payment			
		hours or accessibility			
		type or number of clients served			
		way appointments are offered			
		intake forms			
		signage			
		materials on display			
		other			
	What do you think about these changes?	evaluation of changes			

	Are there been changes that you think should be happening at your agency but aren't?	aspirations/needed change			
Final Thoughts	Is there any other information that you think is important for us to know that hasn't been covered in this interview?	summary/additional info			
Other Observations	-	-			
Important Quotations	-	-			
